# Supplementary material for: MetaRibo-Seq measures translation in microbiomes
Source: Nat Commun. 2020 Jun 29;11:3268. doi: 10.1038/s41467-020-17081-z (PMC7324362; doi:10.1038/s41467-020-17081-z)
Supplement: Supplementary file 10 — Supplementary Data 7 [file 41467_2020_17081_MOESM10_ESM.zip › File2/Confidence_High_Taxonomy/436072_out.krona.html]

Javascript must be enabled to view this page.

members
magnitude
magnitudeUnassigned
count
unassigned
taxon
rank

436072\_out

40

2
superkingdom
40

40
976
phylum

40
200643
class

171549
order
40

2
815
family

2
genus
816

1739517
species
1

SRS1041138\_contig\_number\_1649

1

SRS017701\_contig\_number\_18974
species
1263054

171552
family
38

1
838
genus


SRS077589\_contig\_number\_2520
1
1263103
species

577309
genus
37

454154
species

SRS013687\_contig\_number\_17223SRS016381\_contig\_number\_148SRS017307\_contig\_number\_32362SRS023914\_contig\_number\_29043SRS045004\_contig\_number\_16813SRS045645\_contig\_number\_24139SRS048164\_contig\_number\_31559SRS049712\_contig\_number\_9718SRS049896\_contig\_number\_15765SRS063370\_contig\_number\_12091SRS063985\_contig\_number\_29163SRS077392\_contig\_number\_29503SRS101376\_contig\_number\_24620SRS1041031\_contig\_number\_30465SRS1041118\_contig\_number\_15598SRS1041140\_contig\_number\_11468SRS105082\_contig\_number\_11579SRS143070\_contig\_number\_10813SRS143876\_contig\_number\_17006SRS147653\_contig\_number\_710SRS893252\_contig\_number\_15110
21

454155
species
4

SRS044535\_contig\_number\_23384SRS077641\_contig\_number\_22995SRS1041134\_contig\_number\_6640SRS146764\_contig\_number\_22103

12

SRS013521\_contig\_number\_12522SRS014534\_contig\_number\_contig-100\_34.35SRS015782\_contig\_number\_40019SRS017433\_contig\_number\_16292SRS050026\_contig\_number\_8214SRS053649\_contig\_number\_12074SRS1041037\_contig\_number\_5160SRS1041130\_contig\_number\_4995SRS1041143\_contig\_number\_6460SRS1054928\_contig\_number\_5698SRS145497\_contig\_number\_16304SRS147271\_contig\_number\_14254
species
1263095
